# A Case Report of Subtle EKG Abnormalities in Acute Coronary Syndromes Indicative of Type One Myocardial Infarction

**DOI:** 10.21980/J8W06X

**Published:** 2023-04-30

**Authors:** Paige Matijasich, Patrick Bruss, Gregory Reinhold, Zachary Koppelmann

**Affiliations:** * University of Toledo College of Medicine and Life Sciences, College of Medicine, Toledo, OH; ^ ProMedica Monroe Regional Hospital, Department of Emergency Medicine, Monroe, MI

## Abstract

This case report discusses a patient who had subtle EKG abnormalities that were indicative of a pathological amount of coronary artery disease resulting in occlusion of the right coronary artery (RCA) even though ST-elevation criteria for STEMI were not initially present. In the proper clinical setting, focal repolarization abnormalities in conjunction with cardiac risk factors and cardiac symptoms may indicate a high probability of a pathological amount of coronary disease that warrants emergent intervention. We report a case of a 54-year-old male with cardiac risk factors and cardiac symptoms who presented to the emergency department (ED). Initial EKG, while technically abnormal, was not diagnostic. The point of care troponin was elevated at 0.10 ng/mL. Patient ultimately went to the catheterization lab where he was found to have an occlusion of the RCA and left circumflex artery which required stenting.

**Topics:**

Electrocardiogram, ECG, cardiology, myocardial infarction.

**Figure f1-jetem-8-2-v1:**
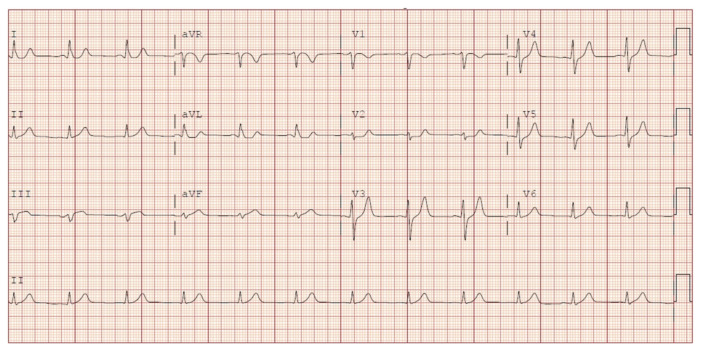


**Figure f2-jetem-8-2-v1:**
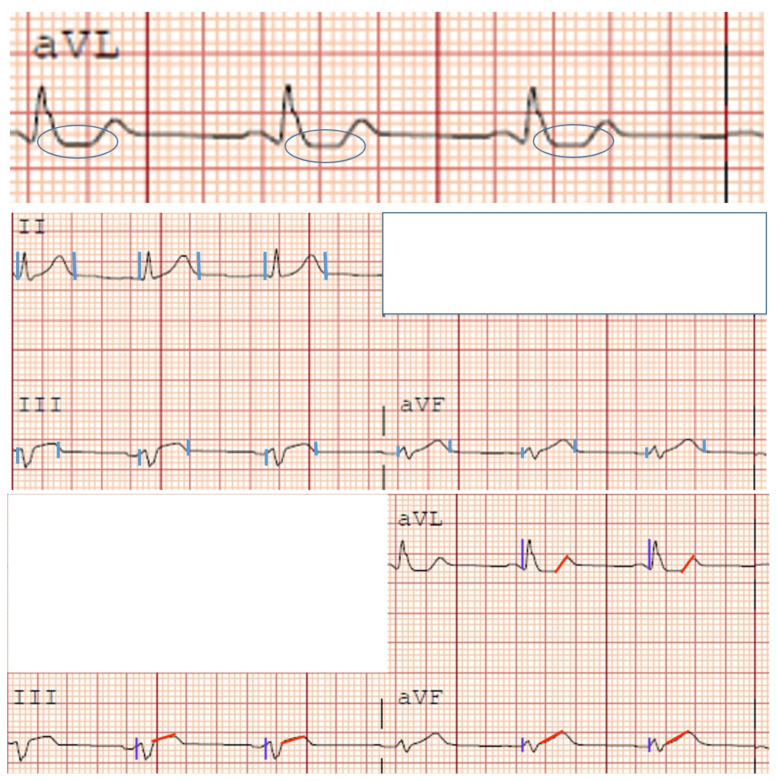


## Brief introduction

[Fig f1-jetem-8-2-v1][Fig f2-jetem-8-2-v1]Cardiovascular diseases (CVDs) are a leading cause of death globally, with ischemic heart disease (IHD) and stroke primarily contributing to this mortality and morbidity.^1^,^2^ CVDs consist of diseases such as ischemic heart disease, stroke, heart failure, peripheral arterial disease, and other cardiac and vascular conditions.^3^ Although some studies suggest CVD and IHD mortality rates are falling, IHD still remains the single greatest contributor to mortality and is a major threat to public health.^4^,^5^ Ongoing research about diagnostic and treatment protocols for acute coronary syndromes is needed in order to better address acute myocardial infarctions (AMI) in the emergency setting. There is a relative consensus regarding the five types of clinical acute myocardial infarctions (AMI). A type-1 AMI is when the level of coronary disease becomes sufficient to obstruct or occlude blood flow to the myocardium. Typically, a type-1 AMI has an emphasis on the causal relationship of plaque disruption with coronary atherothrombosis.^6^ Governing bodies such as the American Heart Association (AHA), American Cardiology Society (ACS), the Society for Cardiac Angiography and Interventions (SCAI), and American College of Cardiology create and endorse guidelines for coronary angiography and best practices in the catheterization lab.^7^,^8^ Therefore, the EKG abnormalities that are diagnostic of acute coronary syndromes (ACS) and indicative of emergent catheterization are set forth by these governing bodies. The traditional and most widely used classifications of ACS are ST-segment elevation myocardial infarction (STEMI), non-ST-segment elevation myocardial infarction (NSTEMI), and unstable angina.^9^ However, the current ST-elevation myocardial infarction (STEMI) vs. non-STEMI (NSTEMI) classification prevents some ACS patients from receiving needed emergent catheterization, and thus may benefit from a revamp. Specifically, one study explored how NSTEMI patients with acute coronary occlusion are denied emergent reperfusion, in spite of their known increased mortality compared with NSTEMI without occlusion.^10^ The lack of emergent catheterization is usually due to the patients not meeting the STE elevation criteria. Some studies have further suggested that a more appropriate method of determining emergent catheterization be based on occlusion MI vs. non-occlusion MI. These studies have found that patients who present with NSTEMI but have total occlusion have a longer delay in identification and subsequent worse outcomes compared to those who present with STEMI.^10^,^11^ These findings suggest the need for better risk stratification tools to determine criteria for emergent catheterization and revascularization.

## Presenting concerns and clinical findings

*A* 54-year-old male with a past medical history of obesity, diabetes mellitus, hypertension, and family history of coronary disease on both maternal and paternal sides presents with chest pain. He currently uses tobacco. He has no known history of coronary artery disease and has never had any previous cardiac workups prior to presentation. He describes substernal pressure that radiates to his left arm worse with exertion, namely while working in the yard, and is associated with diaphoresis. The chest pain has been gradually onset over the past 3 days. Initially, it was associated with exertion and relieved with rest, but on presentation his chest pain began at rest and started one hour prior to arrival.

## Significant findings

The ECG does show multiple subtle abnormalities that in conjunction with his symptoms and risk factors are concerning for ischemia and/or occlusion of the coronary artery vessel.

1. ST depression in aVL. Although slight, the ST segment is below the TP segment or isoelectric point (blue circles).
2. Focal hyper QT waves. The T-waves in II, III, AVF V2, V3, and V4 are hyper acute, namely peaked and tall in relationship to the QRS. These are best displayed in leads II, III, and AVF where the T-waves are taller than the QRS amplitude (vertical blue lines).
3. Straightening of the ST segment. Multiple leads display a straight ST segment namely aVL, III, AVF, and V2 (red lines). Of note, the length of the straight ST segment is greater than 1/4 the amplitude of the QRS (purple lines).
4. Although subtle, these abnormalities are focal in nature.

## Patient course

Once the patient arrived to the ED, initial EKG was abnormal, but not diagnostic of acute infarction. Electrolytes on admission were all within normal range. Chest X-ray, AP chest upright with comparison to prior chest X-ray in 2012, showed no acute findings. Point of care troponin was elevated at 0.10 ng/ml which led to the patient’s ultimately going to the catheterization lab. Catheterization showed: 1) No important disease in left main coronary artery, 2) 60% stenosis in left anterior descending artery, 3) No important disease in the ramus intermedius, 4) 95% stenosis of left circumflex artery, and 5) 100% stenosis of right coronary artery (RCA). Cardiology believed the left circumflex artery was the culprit and determined the RCA was likely a chronic total occlusion. A drug-eluting stent was successfully placed from the mid circumflex to the distal circumflex lesion in the left circumflex artery, and a brief attempt of percutaneous intervention of the RCA was done but aborted when the wire would only go down branches. There were no surgical complications, and there was 0% residual stenosis post intervention. The patient was discharged home within 48 hours with no further complications.

## Discussion

For patients that have cardiac risk factors, cardiac symptoms, and focal repolarization abnormalities, we feel this represents a high pretest probability of a pathological amount of coronary artery disease and may warrant an emergent catheterization regardless of not meeting STE criteria. We therefore are interested in using a simple algorithm consisting of symptoms + risk factors + focal repolarization to determine emergent heart catheterization instead of the classic STEMI vs. NSTEMI classification. In this case, the focal repolarization abnormalities noted were the following: 1) ST depression in aVL, 2) Linearity of the ST segments greater than one quarter the QRS in multiple leads, 3) T-wave amplitude approaching or greater than the amplitude of the QRS complex.

The role of early cardiac catheterization (CC) and coronary revascularization in the management of acute coronary syndromes (ACS) remains highly debated. Initial randomized trials failed to show clear reductions in mortality with early invasive strategies for STEMI and NSTEMI.^12^–^14^ However, more recent studies have shown early revascularization to be beneficial for specific high-risk subgroups of patients.^14^ A study in the *American Heart Journal* showed that inhospital cardiac catheterization is associated with lower mortality in high-risk patients after STEMI and NSTEMI.^14^ It is important to note that this same study showed no difference in mortality in low-risk and intermediate-risk patients, but this data supports the hypothesis that patients with the proper high-risk profile and focal EKG repolarizations may benefit from an early invasive strategy regardless of not meeting STE criteria.

Although ST elevations on an EKG are currently considered the main diagnostic tool for acute MIs, there are other predictable changes on an EKG, which paired with clinical symptoms and risk factors, may signal an acute MI. After QT prolongation, hyperacute T waves are the earliest-described electrocardiographic sign of acute ischemia, preceding ST-segment elevation.^15^ Hyperacute T waves are most evident in the anterior chest leads and are usually present up to 30 minutes after the onset of the infarction.^16^ After these hyperacute T waves, and before the clinically relevant ST segment elevations, the ST segment may actually straighten, with loss of the ST-T wave angle.^16^ These subtle changes which occur before or without STE may clue physicians in on possible AMIs and allow for more prompt treatment, thus improving reperfusion and decreasing mortality.

The criteria set forth by governing bodies for an ST-elevation myocardial infarction that indicate the need for emergent catheterization are not the only reasons a patient may need emergent intervention. Purely focusing on STE criteria may delay the revascularization needed to prevent morbidity and mortality in patients with acute coronary syndrome. We believe serial enzymes, echocardiogram, or stress testing would not be sufficient to rule out possible life-threatening pathologies, and thus there should be a low threshold for left heart catheterization in patient populations presenting with risk factors, symptoms, and focal EKG repolarization abnormalities.

## Supplementary Information








