# Supplementary figures and images for: A Case Report of Subtle EKG Abnormalities in Acute Coronary Syndromes Indicative of Type One Myocardial Infarction
Source: J Educ Teach Emerg Med. 2023 Apr 30;8(2):V1–5. doi: 10.21980/J8W06X (PMC10332673; doi:10.21980/J8W06X)

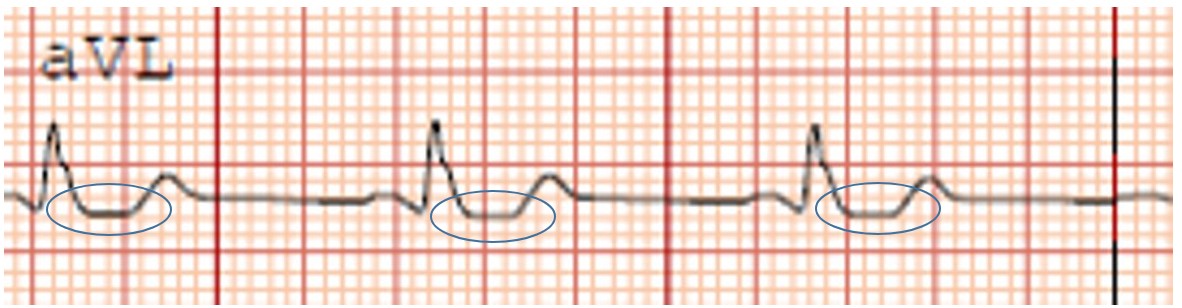

Supplement: Supplementary file 1 [file JETem-8-2-V1-supp1.jpg]

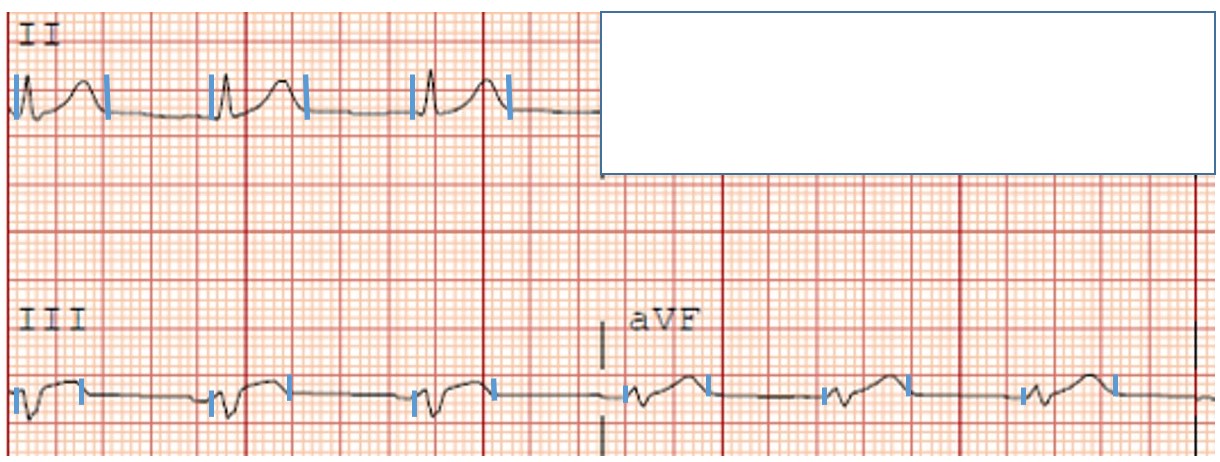

Supplement: Supplementary file 2 [file JETem-8-2-V1-supp2.jpg]

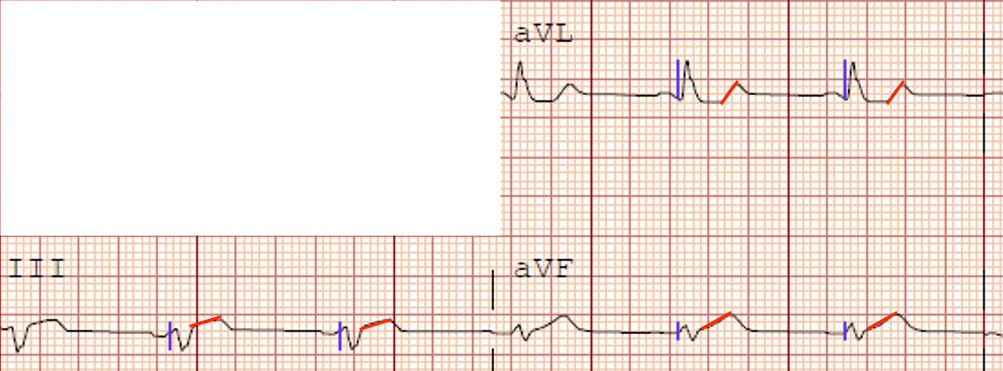

Supplement: Supplementary file 3 [file JETem-8-2-V1-supp3.jpg]

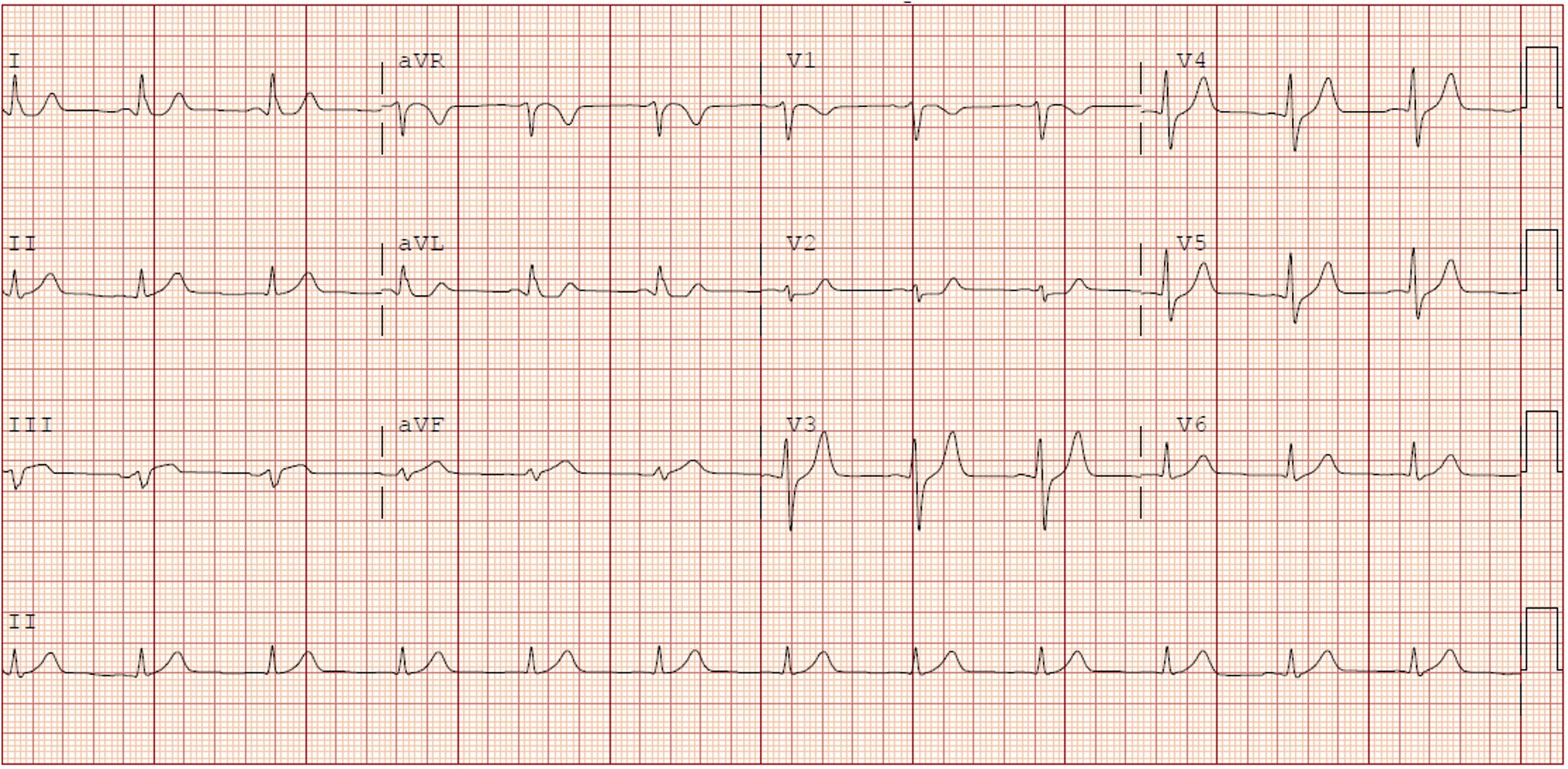

Supplement: Supplementary file 4 [file JETem-8-2-V1-supp4.jpg]
